# “Writing nutritionistically”: A critical discourse analysis of lay people’s digital correspondence with the Swedish Food Agency

**DOI:** 10.1177/13634593211038533

**Published:** 2021-09-20

**Authors:** Karolin Bergman, Paulina Nowicka, Karin Eli, Elin Lövestam

**Affiliations:** Uppsala University, Sweden; University of Oxford, UK; Uppsala University, Sweden

**Keywords:** food, healthy eating, lay communication, nutritionism, official dietary guidance

## Abstract

This article analyzes lay people’s use of nutritionistic discourse in written correspondence with the Swedish Food Agency, an authority responsible for dietary advice. Examining 60 food related written digital messages, we apply a critical discourse analysis to parse the lexical items and grammar people use when constructing “food” in scientific terms. The findings show how message writers place nutrients at the discursive center. Message writers’ grammatical constructions instrumentalize food and eating. This is reinforced by the message writers’ frequent use of terms that indicate preciseness, such as numbers and amounts. Messages therefore emphasize the what, but not the how, of eating, implying a focus on food as subject to regulation and control. As such, eating is discursively reduced to an act of ingesting nutrients that can be decontextualized and managed in isolation—as entities to increase or avoid separately. These discursive features preclude the conceptualization of food choice and eating as subjective experiences of feelings, taste, and tradition.

## Introduction

In texts ranging from government-issued dietary guidelines to popular media articles, healthy eating is discursively framed through a nutricentric approach to food. Extracting nutrients—referring to substances needed for human growth and bodily functions—and the biochemical composition of food from wider contexts of eating and tasting, these discourses portray healthy eating as achieved individually, through the selection of particular combinations of macro- and micro-nutrients. Scrinis (2013) has termed this reductive understanding of food “nutritional reductionism,” or “nutritionism.” Critiques stress that an exclusive nutritionistic approach brings with it an ignorance of traditional, cultural, sensual, and ecological aspects of food and eating that can also affect individuals’ food related well-being (Scrinis, 2013). In this paper, we examine how nutritionistic discourses are manifested in lay peoples’ written communication about healthy eating with a food authority responsible for dietary guidelines. Our research is premised on the idea that discourse and social structure are connected, with discourses participating in the construction of systems of knowledge and belief (Fairclough, 1992). Social representations of healthy eating are therefore determined by discursive structures of dominant discourses (van Dijk, 1993). It is important to identify and characterize these discursive structures and their social consequences, as well as the underlying relations of power that inform them, in order to understand when certain discourses on healthy eating promote wellbeing, and when these discourses constrain understandings of healthy eating and the practices thereof (Fairclough, 1993).

To date, most analyses of nutritionism have focused on United States (US) policy discourses and food industry practices. In these analyses, Scrinis (2013, 2008) defined nutritionism as an ideology and social theory of nutrition, whereas Mudry (2010) identified the quantification of food and nutrition as a key characteristic of nutritionistic rhetoric. Scrinis (2013) has defined three types and “eras” of nutritional reductionism, each driven by scientists, government agencies, public health institutions, and the food industry. The first era, “Quantifying nutritionism,” focused on quantities and proportions of nutrients. The second era, “Good and bad nutritionism,” focused primarily on nutrients to avoid. The third era, “Functional nutritionism,” focused on particular nutrients as contributing to bodily health. In all three eras, nutritionism has incorporated a logic of calculation and quantifying, promoting understandings of food as a substance to be managed through standardized measures (Mudry, 2010; Scrinis, 2008).

Nutritionism has become self-evident in scientific and policy discourses, as well as in popular media publications (Dodds and Chamberlain, 2017; Fairclough, 1992; Mayes and Thompson, 2015; Scrinis, 2008). This discursive embeddedness of nutritionism has encouraged people to behave as “nutricentric persons”: subjects who apply a “nutritional gaze” to themselves and to the foods they consume, conceptualizing food as a “collection of nutrients,” and using nutritional concepts and categories to measure, monitor, and manage their dietary intake (Scrinis, 2013).

### Dietary advice in a nutricentric society

Dietary guidelines are formulated through a discourse of nutritionism. Since the beginning of the 20th century, official dietary advice has been developed through a nutricentric and quantitative approach (Mudry, 2010). In the US, the first official dietary guidelines were based on Atwaters’ chemical findings of macro-nutrients, with calories, instead of food, positioned at the center of attention (Austin, 1999). In the Global North, nutritional science became integrated into governance, with scientific advances—for example, the discovery of vitamins—propelling official dietary standards and advice (Coveney, 2006; Mudry, 2010; Qvarsell, 2005; Santich, 2005). Thus, while official dietary advice might claim neutrality through invoking quantities and nutrients (Bonotti, 2015), it actually reflects historical and societal constructions of knowledge, expertise, good citizenship, and governance (Crotty, 1995; Mudry, 2010; Nestle, 2013). For example, in the early 1900s, when researchers could define macro- and micro-nutrients in foods, healthy eating advice was based on the logics of food components, with food as the energy source for the body’s physical needs. A “good” housewife was accordingly expected to have nutritional knowledge to provide the family with proper food (Mudry, 2009).

More recently, the WHO has promoted official guidelines focused on food rather than the technical language of nutrition. These guidelines conceptualize diets as made up of foods, rather than the sum of their nutrients, and acknowledge that “consumers think in terms of food rather than of nutrients” (World Health Organization, 1998: 2). Such Food-Based-Dietary-Guidelines (FBDGs), which are now implemented in most European countries, categorize food into five to six groups whose relationship is visualized, most often, through a pyramid shape (Montagnese et al., 2015). Moreover, some recently published FBDGs frame food through a social lens; for example, national dietary guidelines published in Brazil and Canada incorporate social and cultural aspects of eating, with the Brazilian guidelines advocating a return to whole and local foods in place of processed foods (see Health Canada, 2019; Ministry of Health of Brazil, 2014). This could be interpreted as a step away from a nutritionistic approach. Nonetheless, most FBDGs continue to employ quantification and technical terms (Kromhout et al., 2016); these features of nutritionistic discourse are also endorsed by food industry and other stakeholders, both in engaging with official dietary advice and in marketing foods (Bergman et al., 2018; Scrinis, 2016). The nutrient focused and bio-chemical aspects of food implicate important aspects of nutritional wellbeing, which remains the main purpose of FBDGs (WHO 1998). Indeed, nutrient focused dietary advice has benefited public health over the last century, illuminating how specific nutrients relate to health and illness (Kiple and Ornelas, 2000).

Sweden’s latest dietary guidelines were published in 2015. When the Swedish Food Agency formulated the guidelines, it was primarily informed by scientific evidence concerning health aspects and environmental impact, as well as by Swedish food culture and tradition, and by the likelihood that people will follow the advice. To ensure that the public could easily understand the guidelines, when advice was formulated, it was tested with members of the public with regard to comprehensiveness, tonality and appeal, form language, and image choice (Brugård Konde et al., 2015). However, despite the focus on food that many dietary guidelines now employ, the narrow and reductive conceptualization of healthy eating represented by nutritionism continues to dominate national dietary advice, including Sweden’s. This is problematic because nutritionism sidelines other perspectives, especially qualitative ones, on what healthy eating means (Mudry, 2009; Scrinis, 2008, 2013).

### Nutritionism in lay discourses

Among lay people, healthy eating is now commonly conceptualized on the level of nutrients (Ditlevsen et al., 2019; Potter et al., 2016). However, to our knowledge, only two studies have analyzed lay peoples’ use of nutritionistic language on the topic of healthy food. Both studies analyzed written communication in internet discussions and blogs in Finland. Jauho (2016) found that lay people who argue for Low-Carb/High Fat (LCHF) diets endorse the scientific language of nutritional recommendations. They also argue that health is physiologically affected by nutrients and that “incorrect” diets can cause disease. Similarly, Finnish bloggers writing about healthy eating use the abstract terms of nutritional science and give quantified advice. By citing experts, research studies, and nutrient measures, these bloggers give credibility to their claims, connecting scientific discourses to anecdotes of their own personal experiences (Huovila and Saikkonen, 2016). In Sweden, the “LCHF” diet is popular as well, and advocates of this diet, as well as influential “LCHF” bloggers, likewise engage in internet debates on the “right” composition of nutrients for dietary health, using scientific discourses to express critique of nutrition authorities and recommendations (Gunnarsson and Elam, 2012). A previous study has found that, in writing, lay people in Sweden use scientific discourses similar to those employed by the Swedish Food Agency (Bergman et al., 2019). This study, alongside the studies on “LCHF” proponents and followers, demonstrates the scientification of food in lay discourses in Scandinavia. However, the detailed linguistic construction of these discourses has not yet been addressed in lay communication or in previous analyses of nutritionism and quantitative discourse (see e.g. Mudry, 2009; Scrinis, 2008, 2013).

In the present study, we analyze lay people’s discursive constructions of nutrition and healthy eating in written correspondence with the Swedish Food Agency, an authoritative body responsible for dietary advice. In this context, nutritionistic discourse is explicitly present, allowing us to delineate its linguistic and discursive features. In turn, this provides a template for the discerning and deconstruction of nutritionism in other societal arenas, such as everyday media, blogs, or advertisements, rendering otherwise self-evident or “hidden” nutritionistic discourses visible. Such detailed awareness of the discursive structures of nutritionism allows for reflection over often unseen linkages between healthy eating discourses, social processes and power relations (Fairclough, 1993), and might open up possibilities for other (perhaps more favorable) discourses to enter the field. One example is the discourses of individualization and blame identified by Törrönen and Tryggvesson (2015) in their analysis of Swedish public health communication about alcohol during pregnancy. Through critically analyzing these individualizing discourses, the authors suggest, broader discourses of societal responsibility and support for the prevention of alcohol abuse may enter the field. Our previous study has found that people use scientific discourses when corresponding with the Swedish Food Agency (Bergman et al., 2019); this correspondence, therefore, provides a particularly rich dataset for an analysis of lay nutritionistic discourses. Examining this correspondence, we parse the lexical items and grammar people use when they employ nutritionism, elucidating how nutritionistic concepts are embedded in everyday discourses on healthy food and healthy eating.

## Methodology

### Material and data collection

This study is part of a doctoral research project on dietary advice and conceptualizations of healthy eating and authority. The project analyzes correspondence with the Swedish Food Agency, specifically, digital messages (by e-mail or web forms) sent from lay people to the Agency, all of which are stored in an Agency database. In an earlier study, we analyzed a corpus of 727 messages from this database and found that lay communications used nutrient- and scientifically-focused discourses similar to those employed by the Agency (Bergman et al., 2019). In the present study, we use a smaller sample from the same corpus of correspondence to carry out a deeper and more detailed analysis of nutritionistic discourse, with specific focus on messages where nutrients were mentioned.

We collected digital messages during the second half of 2016 (1 July–31 December 2016). These messages were collected from a Swedish Food Agency database where all incoming correspondence with the public is categorized and stored. Messages were sorted by Agency personnel according to the following subjects: “Control and regulation,” “Food and health,” “Opinions and comments,” and “Other.” In 2016, the total number of incoming messages stored in the database was approximately 6900, of which slightly less than 50% was categorized under “Food and health,” the category of interest for this study. Messages were chosen for analysis from the sub subgroup “General dietary advice” which was found in the subgroup “Nutrition.” Choosing this sub-subgroup allowed us to capture messages on the topic of everyday eating; the other sub-subgroups (11 in total) focused on more specific topics such as allergies, preschool, and nutritional value. Messages submitted by professionals employed in food-related fields, students, and scientific experts were excluded, as were messages concerning certain medical conditions and technical questions about the website. This process resulted in 60 food-related messages, all of which were included in the “General dietary advice” sub-subgroup and submitted by lay people. These messages ranged from 11 and 264 words with a median of 61 words per message (greetings excluded).

### Analyzing computer mediated communication

Digital messages are particularly informative for our discourse analysis, as online communication is now the central means by which members of the lay public contact government authorities in Sweden. Sending digital messages or questions to an authority is a social practice akin to letter-writing (Barton and Hall, 2000). Swedish Authorities are obliged by law to respond promptly and give information and advice to individuals who request it^1^ (Justitiedepartementet, 1986). Emails and digital text entries are nevertheless asynchronous modes of communication, due to the temporal and spatial distance between sender and recipient (Beer, 2017). While sending a digital message may seem informal and involve little effort, writing such a message gives senders the opportunity to organize and articulate their thoughts (Beer, 2017). However, sentence fragmentation and incomplete stylistics, such as missing punctuation or incorrect capitalization, are common in emails. Because of this, “utterances,” in place of “sentences,” can be considered the basic unit in computer mediated discourse. Emails are commonly composed of many utterances which create an internal structure in the text (Herring and Androutsopoulos, 2015). For example, in the present study, emails and text entries commonly have a structure which starts with a greeting, followed by a query and a closing greeting.

### Theoretical approach: Critical discourse analysis

To study the linguistic features of nutritionistic discourse, we draw on principles of Critical Discourse Analysis (CDA) and the stance that discourses have constitutive effects that contribute to the construction of systems of knowledge and belief. Language use is thus seen as a form of social practice (Fairclough, 1992). Theoretically, we take a social constructionist position whereby knowledge is understood as socially constructed and culturally and historically mediated (Burr, 2015). In Fairclough’s three-level model of Critical Discourse Analysis (1992) the *text* is the smallest grammatically constructed unit which always relates to discursive and social practices. The *discursive practice* concerns the setting around the text, where and how it is produced and how it builds on other texts. The discursive practice is embedded within broader *social practices*. Throughout, relations of power influence which discourses become dominant. Although the reproduction of discursive dominance may be “naturalized,” and hence hidden, in everyday situations, following a CDA approach allows us to elucidate relations of power as they shape access to discourses and discursive influences on knowledge, understanding, and values (van Dijk, 1993).

Our aim is to analyze healthy eating as a social object in a system of knowledge and belief. Our analysis focuses on how “food” is constructed in scientific terms by lay people in correspondence to the Swedish Food Agency. To analyze this correspondence, the texts were first carefully and repeatedly read, looking for discursive patterns. Recurring terms and themes concerning food and eating were recorded and categorized for closer examination. To explain the patterns we had found, we made use of some concepts from functional grammar as conceptualized by Halliday et al. (2014). Our patterns could be explained and characterized by modal auxiliary verbs (verbs that express ability, possibility, permission or obligation, e.g. shall), nominalizations (verbs modified into nouns, e.g. intake), and passive verb constructions (verbs turning objects of an action into the subject of a clause, e.g. milk drinking). We also noted similarities and differences in clause structure—objects, subjects and adjectives, and expressions of quantification. We analyzed the texts in the original Swedish; the example quotes used in the findings section were translated into English for the purposes of this manuscript.

## Findings

The findings identify the main discursive and linguistic features that characterize nutritionistic discourse, as present in digital correspondence between lay people and the Swedish Food Agency. We found that nutrients are discursively centered as subjects or objects. This is combined with a frequent use of terms that indicate preciseness, such as numbers and amounts, and reinforced by modality (auxiliary verbs) and transitivity (nominalizations). Taken together, these discursive devices characterize a nutritionistic discourse of healthy eating.

We have selected four extended texts (Box 1, examples 1–4) to illustrate these discursive traits in detail. Through the extended texts, we place discursive traits into a fuller context, thereby offering a nuanced understanding of how and where these traits are used. These discursive traits can also be found in most of the 60 messages included in our dataset. Therefore, in some instances, we use shorter quotes from the wider corpus to exemplify our findings further (examples 5–10). Throughout this section, in each example quote, we use boldface to indicate words, phrases, and constructions of relevance.

**Box 1.** Examples from the database of incoming correspondence to the Swedish Food Agency.

Example 1: *Hi and thanks for your reply on canned sardines that I had a question about!* *Now I have another question! The case is that I make a big bowl of soup at home with approximately 14 vegetables and leguminous plants and live of this all the time and eat it every day, big portions, two times per day!* *Since one are supposed to eat lunch and dinner (as I am active and practice weight lifting three times a week) therefore I eat two portions of this soup every day. But would it be enough with only one portion per day instead since it is the same food?? Should one have like cottage cheese or protein for dinner instead??*
Example 2: *Hi!* *I am 20 years old and lately I have started to think a lot about my sugar intake. I have never had any problems with high intakes of sugar, but lately I have noticed that there is sugar in most of the things we eat, unfortunately.* *I am active every day, biking, walking, weight lifting, and sometimes running. Therefore I try to have a lot of protein and vegetables/vitamins.* *To my question*: *One of the easiest ways of having protein is to drink milk, large quantities. One liter of “medium fat” milk gives 35 g of protein which is good, but unfortunately it also gives 49 g of sugar. How dangerous is the sugar you get from [Brand name] “medium fat” milk, does it affect my body a lot negatively if I have 50–75 g sugar from milk every day?*
Example 3: *Hello, My name is [Name] and I have two important questions to you concerning recommendations about*: *1. One has read both this and that about eggs and that it can affect cholesterol negatively. . . Now I want to know if one can have one egg per day without risk of this. Can one even have two a day—always?* *2. This question concerns carbohydrates. A woman around 65-years. Live a relatively active life and eat healthy and varied. BUT excludes carbohydrates to the meals; eat for example fish/meat/chicken and a salad with oil to that. On the other hand she eats two fruits per day and a smoothie with berries in the morning. Is it okay to exclude carbohydrates in this way? I have read that carbohydrates are needed for functions in the brain. . . I have also heard that one even NEED carbohydrates to lose weight (she wishes to lose 3–4 kilos). Is it possible to build muscles without carbohydrates to every meal—She does have fruit every day. . . is it enough?*
Example 4: *Is it true that a high intake of milk increases the risk of cancer? Many speak about it in example here: [You Tube-link] I drink approximately 1 liter of milk every day. [link to webpage] This study says that a high intake of dairy products can increase the risk of prostate cancer among other. Should I decrease my drinking of milk? I also eat approximately 0.3 l of sour milk every day. Should I be worried?*

### Nutrients and foods discursively centered as objects and subjects in utterances

Message writers frame nutrients as the key characteristics of food and as the main topic of interest in inquiries about healthy eating. Grammatically, this focus on nutrients is accomplished through utterances where a nutrient functions as an object, subject, or adjective. In other instances, nutrients are emphasized through references to foods as carriers of nutrient components.

#### Nutrients as objects

In many of the messages, a nutrient or nutrient group is mentioned as the direct focus of the writer’s question and, grammatically, as the object in an utterance. For example, one message writer introduced their message as follows: “*I am a recent vegan and have a question about omega-3 and fats in general. . .*” (example 5). Example 3 in our selected texts has a similar structure where carbohydrates are the direct object of the writer’s question:

*This question* ***concerns carbohydrates*** *. . . eat healthy and varied. BUT* ***excludes carbohydrates*** *to the meals. . .*

Many of the writers ask about what constitutes a sufficient or optimum intake of particular nutrients, thereby framing healthy eating as instrumental—as having one main function of providing the eater with an optimal amount of nutrients. Nutrients, then, are placed as objects when writers describe what they eat or ask about what to eat. In example 2, both protein and sugar (as carbohydrate) are mentioned:

*One of the easiest ways of* ***having protein*** *is to drink milk, large quantities. One liter of “medium fat” milk* ***gives*** *35 grams of* ***protein*** *which is good, but unfortunately it also* ***gives*** *49 grams of* ***sugar***.

As in the example above, many messages mention food items together with their key macro-nutrient as the source that provides their “content”; however, in other messages, a nutrient may be compared with a food product in order to characterize it. In example 1, a macro-nutrient (protein) is used interchangeably with cottage cheese:

*. . .would it be enough with only one portion per day instead since it is the same food?? Should one* ***have*** *like* ***cottage cheese or protein*** *for dinner instead??*

In this example, the writer mentions protein as a food one could have for dinner. The writer’s question about eating one or two portions of the same food per day implies that the macro-nutrient content of a single food, rather than the composition of the meal as a whole, is more important.

In example 3, the writer describes excluding carbohydrates from one’s diet, and then goes on to equate fruit with carbohydrates:

*Is it possible to build muscles* ***without carbohydrates*** *to every meal – She* ***does have fruit*** *every day. . . is it enough?*

Fruit, in this example, is characterized by a single nutrient, with the nutrient in focus giving value to the food as its carrier.

#### Nutrients as subjects

While less common than messages that place nutrients as objects, some messages also place nutrients as subjects. For example, in one message (example 6), the writer offers an interpretation of a state representative’s dietary advice, as presented on a television program: “*. . .* ***saturated fats should be avoided*** *due to the risk of be taken with cardio-vascular disease.*” Here, the nutrient is the subject, while the person who is supposed to avoid it is not mentioned at all.

In some messages, nutrients are described as agents, which in themselves are capable of achieving things. In these constructions, the nutrient is the subject of the utterance accompanied by a predicate which describes the nutrient’s “action,” for example: “*. . . it is all these* ***carbohydrates*** *and especially fast ones and sugar in various forms that* ***make*** *people fat and sick*.” (example 7).

#### Foods as objects and subjects

As with nutrients, some writers position foods as active entities separate from wider contexts of eating and meals. In example 3, which focuses on the risk of consuming eggs, eggs figure discursively as both subject and object:

*one has read both this and that about* ***eggs*** *and that* ***it can affect*** *cholesterol negatively. . . Now I want to know if* ***one can have one egg*** *per day without risk of this.*

In the first part of the question, “eggs” is a subject with agency to act upon the body (through raising cholesterol). In the second utterance, “egg” is the object in the interrogative clause. In both these constructions, the “egg” is separated from contexts of eating and discussed reductively in relation to bodily health.

### Preciseness and control

In many messages, writers use numbers, calculations, and expressions of quantitative restriction and formality when referring to food and nutrients. Eating is thereby discursively reduced to an act of ingesting nutrients, and food is framed as a substance to be controlled through quantitative measures.

#### Use of numbers

When asking for or reflecting on dietary advice, writers often frame this advice in quantified terms, asking how much one can eat and where the numbered limits are set. For instance, in example 4, a writer quantifies their own milk consumption, to be assessed by the Swedish Food Agency as “healthy” or “unhealthy”:

*I drink approximately* ***1 liter*** *of milk every day. . .. Should I decrease my drinking of milk? I also eat approximately* ***0.3 liters*** *of sour milk every day.*

Some writers also include their own calculations of nutrient intake when asking how much they can eat of a certain food, as in example 2, where grams of protein are calculated against grams of sugar to decide whether milk is “healthy” or not:

*One liter of “medium fat” milk gives* ***35 grams*** *of protein which is good, but unfortunately it also gives* ***49 grams*** *of sugar. How dangerous is the sugar you get from [Brand name] “medium fat” milk, does it affect my body a lot negatively if I have* ***50-75 grams*** *sugar from milk every day?*

#### Use of adjectives to describe relative amounts

Writers also refer to quantities in relative terms, compared to an ideal amount. Amounts of nutrients or foods are presented as potentially problematic, producing risk through, for example, “high” or “too high” intakes, “much” or “too much” consumption, or “large” or “small” quantities. In some examples, amounts are also spoken about in comparisons to others. The use of these adjectives presupposes some kind of “ideal” or “standard” amount to which the amount in question is compared, as in example 2, which problematizes sugar consumption:

*I have never had any problems with* ***high intakes of sugar***, *but lately I have noticed that there is sugar in most of the things we eat, unfortunately.*

When the adjectives used specify excessive quantities, they are often associated with action verbs that invoke reduction, for example, “keep them down,” as in the following quote about carbohydrates: “*If one wish to keep GI [glycemic index] as low as possible. . . should one then keep the CONTENT OF CARBOHYDRATES or the CONTENT OF SUGARS down*. . .” (example 8). Another example is the verb “decrease,” which is used example 3:

*Is it true that a* ***high intake of milk*** *increases risk of cancer? Many speak about that. . . . . . I drink approximately 1 liter of milk per day. . . Should I* ***decrease*** *my drinking of milk? I eat approximately 0.3 liters of sour milk per day as well. Do I need to worry?*

#### Implications of rules and restrictions by use of “modal auxiliary verbs.”

Writers convey conceptualizations of food and eating as embedded in certain rules and as individually controlled through particular verbs and question formulations. The use of modal auxiliary verbs such as *can*, *shall*, and *should/would* implicates a certain degree of obligation, which in this case is positive—you are to do something. “Can,” “shall,” and “should/would” are used in questions on and description of how to practice healthy eating. These particular verbs (can, shall, and should/would) express obligation in different degree. In example 4, the message writer asks for advice on how to judge his/her habits of drinking milk, using “should”:

***Should I*** *decrease my drinking of milk? I also eat approximately 0.3 liters of sour milk every day.* ***Should I*** *be worried?*

This writer asks for advice on how to restrict consumption of dairy products to reduce risk for cancer. Others ask to be provided with dietary rules to follow, not only by using the auxiliary verbs, but also through the expressions “what about” and “how is it” (“vad gäller” och “hur är det med. . .”). In the Swedish context, these phrases imply that the writer is asking for rules to follow. Many writers also ask how much of a certain food or nutrient is recommended, and even if something is “allowed” or “okay,” for example “*. . .is it okay to use sugar in that context [previously described]?*” (example 9). In this way, message writers frame food and healthy eating as framed by rules and restrictions that call for defined actions.

#### Instrumentalizing the activity of eating through nominalization and passive verbs

Nominalization (verbs turned into nouns) and passive verbs (turning the focus away from the subject in a sentence) convey a sense of mechanizing and detachment from the practice of healthy eating. Even though many message writers use the verb “eat,” they also use circumlocutions for how food or food substances enter the body. The most common is the noun “intake,” a nominalization of a verb. This word is often used when messages refer to official recommendations, mirroring authoritative dietary guideline formulations. In example 4, “intake” is used twice in the context of drinking milk:

*Is it true that a* ***high intake*** *of milk increases the risk of cancer? Many speak about it in example here: [You Tube-link] I drink approximately 1 liter of milk every day. [link to webpage] This study says that a* ***high intake*** *of dairy products can increase the risk of prostate cancer among other. Should I decrease my* ***drinking of milk*** *? I also eat approximately 0.3 liters of sour milk every day.*

This message writer uses the nominalized expression “intake” alongside “eat” and “drink” in the description of daily routines. The verb “eat” is used together with a specific quantified amount. When requesting advice, the message writer uses a passive construction and another nominalization: “drinking of milk” (mjölkdrickande).

In other messages, a common alternative to “intake” is the use of the Swedish expression “få i sig,” a passive formulation that means “get [something] into yourself.” In example 2, the message writer describes consuming a food group and nutrients using this phrase:

*I am active every day, biking, walking, weight lifting and sometimes running. Therefore I try to have* ***“få i mig” [get into myself]*** *a lot of protein and vegetables/vitamins.*

The expression “få i mig” is used with reference to both micro- and macronutrients in our corpus. Another example is “*How should I think to get enough of Iodine into myself?*” (example 10). Both these expressions—intake and “få i sig” [get into yourself]—mark the nutricentric reduction of eating, and signal an instrumental relationship between food and the body. In some cases in the wider corpus of correspondence, when the verb “eat” is used, it is in combination with a single nutrient, for example “eat protein.”

## Discussion and conclusion

While scientists, governmental bodies and the food industry have driven nutritionistic discourse (Scrinis, 2013), our analysis explores in detail how lay people continuously reproduce and actively use this discourse. Through deconstructing and delineating lay people’s written communication about healthy eating with the Swedish Food Agency, we have identified features that characterize lay nutritionistic discourse. Our findings show that nutrients are being placed in the discursive center using grammatical and linguistic constructions such as nominalizations, auxiliary verbs, and quantification. These discursive features do not as such create the nutritionistic approach, but they do reinforce a nutritionistic worldview by contributing to its formation and legitimization. A reductive focus on nutrients obscures traditional, cultural, and sensual aspects of food eating, and aligns the public’s understanding of food and the body with the language of marketing, especially as it applies to nutritionally engineered products (Scrinis, 2008). This focus on nutrients and their effects can be characterized as a form of health risk avoidance, with specific nutrients discursively linked to specific threats. This medicalization of food and its effects is tightly linked to nutritionism, and this logic is easy to apply when nutrients are separated from wider contexts of eating (Scrinis, 2013).

In their written messages to the Swedish Food Agency, lay people place discursive focus on the nutrient level by constructing utterances where nutrients are placed as objects or subjects. In real life, nutrients naturally almost never occur in isolated form, but in these linguistic constructions, they are treated as if they were isolated from the more complex food item or meal situation. Thereby lay people conceptualize nutrients as if they could be decontextualized and managed in isolation—as entities to increase or avoid, regardless of the complexity of meal situations or practical, emotional, and social implications. Likewise, message writers discursively separate single foods from their wider context. In public health policy discourses, sugar is singled out as a threat to health, and expectations of population health gains from a reduction in sugar consumption are high. Nutritionism has paved the way for social health promotion strategies, such as anti-sugar movements (Throsby, 2018), as well as “nudging,” choice architecture, and food-taxes (Bonotti, 2015). As Throsby (2018) exemplifies with a quote from a 2015 Public Health England report: “Any significant progress to reduce sugar intake would yield benefits.” This formulation mirrors discursive features found in our data, including the use of the nominalization “intake” and the positioning of sugar as the object to be quantitatively reduced. Similar formulations of sugar and reduction can also be found in Sweden’s official dietary advice (Livsmedelsverket, 2015).

Quantification, one of the main characteristics of the analyzed correspondence, further reinforces the nutritionistic construction of food as manageable. When amounts are possible to specify discursively, they may seem easier to control and modify, and thereby control perceived risks (Mudry, 2009; Scrinis, 2008). Quantification also has the effect of conveying objectivity and impersonality (Porter, 1996). In the messages we analyzed, when message writers referred to food eaten according to particular quantities or calculations, they framed the body as an object upon which nutrients act, with amounts of nutrients conferring risk to the body by being too little or too much. High amounts of protein and sugar, respectively, were described as “good” or “unfortunate,” alluding to a clear valuation of certain nutrients over others, and to an understanding of healthy eating as a process of adjudicating between nutrients that co-exist within the same food. When several nutrients are valued as good or bad (Scrinis, 2013), and where these nutrients together compose the same food, conflicts, and confusion about how to relate to this food may ensue. The promotion of FBDG by WHO is underpinned by this argument, allowing for a more comprehensive view of food and eating, and thereby avoiding the confusion that the isolation of certain nutrients might cause (World Health Organization, 1998). The subjectivity of eating is effectively ignored by quantification, which allows for comparisons of food on a one-dimensional nutritionistic scale, regardless of what foods are in question (Mudry, 2009). The discursive quantification of eating, moreover, is aligned with the self-tracking trend, wherein health and fitness app users are encouraged to conceptualize their bodies and health through numbers (e.g. calories consumed and calories expanded, heart rate, steps taken), with numbers positioned as more reliable than subjective and bodily experiences (Lupton, 2013).

The use of auxiliary verbs further reinforces the conceptualization of food as a quantifiable combination of nutrients to control. Auxiliary verbs (shall/should/can) have the function of modifying prompts to convey different levels of obligation to obey (Halliday et al., 2014). In our analysis, auxiliary verbs were expected, given that the typical format of communication between lay people and the Swedish Food Agency takes the form of a question posed by a member of the public to be answered by the authority. Auxiliary verbs used do not signal the strongest obligation (compare “must”), but are low and medium on the obligation value scale (Halliday, 2004: 622–623). However, the choice of verbs further consolidates the idea that food needs to be controlled within rules that are dictated by expert authorities, and that the person responsible for enacting this control is an individual: the writer him- or herself, or an imagined general member of the public.

To convey an objective perspective on eating, nominalizations and passive formulations are used to describe the act of eating, where the nominalization “intake” is frequently used together with descriptions of quantified nutrients or foods. Similar use of this nominalization has also been noted in clinical dietitians’ notes, where they describe patients’ “intake” of food (Lövestam et al., 2015). In the messages we analyzed, the use of the word “eating” is mostly limited to descriptions of nutrients ingested. The body is not conceptualized as a subject with its own experiences, but rather as an object that bears the consequences of ingesting varying amounts of nutrients.

Working with a set of messages sent by lay people to the Swedish Food Agency has allowed us to focus on how nutritionism is employed in lay discourses. Following the CDA approach, however, it is important to recognize that while nutritionism is a dominant discourse that influences socially shared knowledge and ideas, access to this discourse is mostly reserved for the privileged (van Dijk, 1993). Highly educated people whose body weight is classified as “normal” are more likely to integrate “healthy lifestyle regimes” into everyday practice, and are also more likely to express interest in health information (Smith and Holm, 2010). We based our analysis on the assumption that message writers have the resources both to adopt a nutritionistic discursive style and to communicate with the Swedish Food Agency. Thus, while information about the message writers’ socioeconomic background was not available, their writing to an authoritative body suggests that these writers had access to certain knowledges and discourses needed for corresponding with the “power elite” represented by the Swedish Food Agency. It is possible that nutritionistic discourses may be employed differently across socioeconomic strata.

Our analysis demonstrates the applicability of CDA techniques for analyzing and characterizing nutritionistic discourse on a linguistic level. Discursive practice is entangled with social practice (Fairclough, 1992), such that discursive features conceptually constitute healthy food and healthy eating. The discursive features analyzed in this paper demonstrate how nutritionistic concepts are embedded in lay discourse, forming a taken for granted, and often unexamined aspect of popular knowledge about food and eating. Having internalized a nutritionistic discourse, message writers, through decontextualization, quantification and instrumentalization, emphasize the what, but not the how, of eating. Discussions of food are therefore reduced to questions about components, implying a focus on food as subject to regulation and control. Delineating these specific discursive traits, as we have done in this analysis, offers opportunities to detect and deconstruct nutritionism in other communicative events, and thereby create openings for diverse discourses to frame understandings of health, food, and eating.

## Supplemental Material

sj-docx-1-hea-10.1177_13634593211038533 – Supplemental material for “Writing nutritionistically”: A critical discourse analysis of lay people’s digital correspondence with the Swedish Food Agency Click here for additional data file. Supplemental material, sj-docx-1-hea-10.1177_13634593211038533 for “Writing nutritionistically”: A critical discourse analysis of lay people’s digital correspondence with the Swedish Food Agency by Karolin Bergman, Paulina Nowicka, Karin Eli and Elin Lövestam in Health:

## Notes

1 During data collection the current law version was 1986:223, which later has been replaced by 2017:900.
