# Supplementary material for: “Writing nutritionistically”: A critical discourse analysis of lay people’s digital correspondence with the Swedish Food Agency
Source: Health (London). 2021 Sep 20;26(5):554–70. doi: 10.1177/13634593211038533 (PMC9344565; doi:10.1177/13634593211038533)
Supplement: sj-docx-1-hea-10.1177_13634593211038533 – Supplemental material for “Writing nutritionistically”: A critical discourse analysis of lay people’s digital correspondence with the Swedish Food Agency [file sj-docx-1-hea-10.1177_13634593211038533.docx]

**Quotes in original Swedish**

|  | **Box 1, Examples from the database of incoming correspondence to the Swedish Food Agency, English and original Swedish** |
| --- | --- |
| Example 1: | *Hi and thanks for your reply on canned sardines that I had a question about!*  *Now I have another question! The case is that I make a big bowl of soup at home with approximately 14 vegetables and leguminous plants and live of this all the time and eat it every day, big portions, 2 times per day!*  *Since one are supposed to eat lunch and dinner (as I am active and practice weight lifting 3 times a week) therefore I eat two portions of this soup every day. But would it be enough with only one portion per day instead since it is the same food?? Should one have like cottage cheese or protein for dinner instead??* |
| Example 1 original quote in Swedish: | *Hej och tack för ert förra svar om sardiner på burk som jag hade en fråga om!*  *Nu har jag en till fråga! Det är så nämligen att jag gör storkok-soppa hemma med ca. 14 grönsaker och baljväxter och lever på detta jämt o ständigt o äter det varje dag, stora portioner, 2 ggr/dag!*  *Eftersom att man ska äta lunch och middag (då jag är aktiv och tränar styrketräning 3 ggr i veckan), därför äter jag två portioner av denna soppa varje dag. Men skulle det räcka med bara en portion per dag istället eftersom det är samma mat?? Ska man ta typ kvarg eller protein som middag istället?? (No. U-73233)* |
| Example 2: | *Hi!*  *I am 20 years old and lately I have started to think a lot about my sugar intake. I have never had any problems with high intakes of sugar, but lately I have noticed that there is sugar in most of the things we eat, unfortunately.*  *I am active every day, biking, walking, weight lifting and sometimes running. Therefore I try to have a lot of protein and vegetables/vitamins.*  *To my question:*  *One of the easiest ways of having protein is to drink milk, large quantities. One liter of “medium fat” milk gives 35 grams of protein which is good, but unfortunately it also gives 49 grams of sugar. How dangerous is the sugar you get from [Brand name] “medium fat” milk, does it affect my body a lot negatively if I have 50-75 grams sugar from milk every day?* |
| Example 2 original quote in Swedish: | *Hej!*  *Jag är 20 år och har på senaste tiden börjat ordentligt fundera på mitt sockerintag. Jag har aldrig haft några problem med höga intag av socker men har under senaste tiden märkt att det är socker i det mesta man äter, tyvärr.*  *Jag rör på mig varje dag, cyklar, promenerar, styrketränar och ibland springer. Så därför försöker jag få i mig mycket protein samt grönsaker/vitaminer.*  *Till min fråga:*  *Ett av dem lättaste sätten att snabbt och billigt få i sig protein är att dricka mjölk, stora mängder. En liter mellanmjölk ger 35 gram protein vilket är bra, men det ger också tyvärr 49 gram socker. Hur farligt är sockret man får i sig via Gävleortens mellanmjölk, påverkar det min kropp mycket negativt om jag får i mig 50-75 gram socker var dag ifrån mjölk?  (No. U-67211)* |
| Example 3: | *Hello, My name is [Name] and I have two important questions to you concerning recommendations about:*  *1. one has read both this and that about eggs and that it can affect cholesterol negatively… Now I want to know if one can have one egg per day without risk of this. Can one even have two a day – always?*  *2. This question concerns carbohydrates. A woman around 65-years. Live a relatively active life and eat healthy and varied. BUT excludes carbohydrates to the meals; eat for example fish/meat/chicken and a salad with oil to that. On the other hand she eats 2 fruits per day and a smoothie with berries in the morning. Is it okay to exclude carbohydrates in this way? I have read that carbohydrates are needed for functions in the brain… I have also heard that one even NEED carbohydrates to lose weight (she wishes to lose 3-4 kilos). Is it possible to build muscles without carbohydrates to every meal – She does have fruit every day… is it enough?* |
| Example 3 original quote in Swedish: | *Hejsan, Jag heter [Namn] och har två viktiga frågor till er vad ni har för rekommendationer gällande detta:*  *1. man har ju läst både det ena och det andra om ägg och att detta kan påverka kolesterolet negativt… Nu undrar jag om man kan äta ett ägg om dagen utan risk för detta. Kan man till och med äta två om dagen - alltid alltså?*  *2. Denna fråga gäller kolhydrater. En kvinna i 65-års åldern. Lever ett relativt aktivt liv och äter hälsosamt och varierat. MEN utesluter kolhydrater till måltiderna; äter exempelvis fisk/kött/kyckling och en sallad med olja till. Däremot äter Hon 2 frukter per dag samt en smoothie med bär på morgonen. Är det OK att utesluta kolhydraterna på detta sätt? Jag har läst att kolhydrater behövs för hjärnans funktioner… Jag har också hört att man till och med BEHÖVER kolhydrater för att gå ner i vikt (Hon önskar gå ner 3-4 kilo). Kan man bygga muskler utan kolhydrater till varje måltid - Hon äter ju frukt varje dag… Räcker detta? (No. U-67633)* |
| Example 4: | *Is it true that a high intake of milk increases the risk of cancer? Many speak about it In Example here: [You Tube-link] I drink approximately 1 liter of milk every day. [link to webpage] This study says that a high intake of dairy products can increase the risk of prostate cancer among other. Should I decrease my drinking of milk? I also eat approximately 0,3 liters of sour milk every day. Should I be worried?* |
| Example 4 original quote in Swedish: | *Är det sant att ett dagligt högt intag av mjöl ökar risken för cancer? Många pratar om det Tex här: https://youtu.be/a1dsWjNv3b0. Jag dricker ungefär 1 liter mjölk per dag.*  *http://www.care2.com/greenliving/harvard-declares-dairy-not-part-of-healthy-diet.html Denna studie säger att ett högt intag av mejeriprodukter kan öka risken för bland annat prostatacancer. Bör jag minska mitt mjölkdrickande? Jag äter även ungefär 0,3 liter fil per dag. Bör jag vara orolig? (No. U-67796)* |
| Example 5: | *I am a recent vegan and have a question about omega-3 and fats in general…* |
| Example 5 original quote in Swedish: | *[J]ag är nybliven vegan och undrar en sak om omega 3 och fetter generellt... (No. U-72275)* |
| Example 6: | *…saturated fats should be avoided due to the risk of be taken with cardio-vascular disease.* |
| Example 6 original quote in Swedish: | *…mättat fett bör undvikas pga av risken att drabbas av hjärt- och kärlsjukdomar. (No. U-70800)* |
| Example 7: | *… it is all these carbohydrates and especially fast ones and sugar in various forms that make people fat and sick.* |
| Example 7 original quote in Swedish: | *…det är alla dessa kolhydrater och då särskilt snabba sådana och socker i olika former som gör människor feta och sjuka. (No. U-67081)* |
| Example 8: | *If one wish to keep GI [glyceamic index] as low as possible… should one then keep the CONTENT OF CARBOHYDRATES or the CONTENT OF SUGARS down*… |
| Example 8 original quote in Swedish: | *Om man vill ha så lågt GI som möjligt (alltså så lågt insulinpåslag som möjligt) ska man då hålla KOLHYDRATHALTEN nere eller SOCKERART-HALTEN nere… (No. U-72511)* |
| Example 9: | *…is it okay to use sugar in that context [previously described]?* |
| Example 9 original quote in Swedish: | *…är det ok att använda socker i det sammanhanget? (No. U-70792)* |
| Example 10: | *How should I think to get enough of Iodine into myself?* |
| Example 10 original quote in Swedish: | *Hur ska jag tänka för att få i mig tillräckligt med jod? (No. U-72176)* |
